# Supplementary material for: Sleep pressure accumulates in a voltage-gated lipid peroxidation memory
Source: Nature. 2025 Mar 19;641(8061):232–9. doi: 10.1038/s41586-025-08734-4 (PMC12043502; doi:10.1038/s41586-025-08734-4)
Supplement: Supplementary file 1 — Supplementary Tables 1 and 2. [file 41586_2025_8734_MOESM1_ESM.pdf]

nature portfolio

https://doi.org/10.1038/s41586-025-08734-4

Supplementary information

Sleep pressure accumulates in a voltage-gated lipid peroxidation memory

In the format provided by the authors and unedited

Supplementary Table 1. Statistical analyses of data in Figures 3–6.

Figure	Statistical test	Pairwise comparison	Test statistic	*P*
**3a**	Two-way repeated-measures ANOVA			
	Zeitgeber time		$F_{47,5029}$ = 115.1	<0.0001
	Genotype		$F_{3,107}$ = 57.28	<0.0001
	Zeitgeber time × Genotype		$F_{141,5029}$ = 11.90	<0.0001
	Holm-Šídák test	*+/Y* vs. sni^1/Y	t_{107} = 12.31	<0.0001
	Holm-Šídák test	*+/Y* vs. *nSyb-GAL4 > UAS-AOX* (sni^1/Y)	t_{107} = 1.909	0.0589
	Holm-Šídák test	*+/Y* vs. *R23E10-GAL4 > UAS-AOX* (sni^1/Y)	t_{107} = 3.685	0.0007
	Holm-Šídák test	sni^1/Y vs. *nSyb-GAL4 > UAS-AOX* (sni^1/Y)	t_{107} = 9.438	<0.0001
	Holm-Šídák test	sni^1/Y vs. *R23E10-GAL4 > UAS-AOX* (sni^1/Y)	t_{107} = 7.195	<0.0001
**3b**	Two-way repeated-measures ANOVA			
	Zeitgeber time		$F_{47,5781}$ = 103.5	<0.0001
	Genotype		$F_{3,123}$ = 44.64	<0.0001
	Zeitgeber time × Genotype		$F_{141,5781}$ = 10.42	<0.0001
	Holm-Šídák test	*+/Y* vs. sni^1/Y	t_{123} = 10.76	<0.0001
	Holm-Šídák test	*+/Y* vs. *nSyb-GAL4 > UAS-*Hk^{RNAi} (sni^1/Y)	t_{123} = 4.636	<0.0001
	Holm-Šídák test	*+/Y* vs. *R23E10-GAL4 > UAS-*Hk^{RNAi} (sni^1/Y)	t_{123} = 1.507	0.1344
	Holm-Šídák test	sni^1/Y vs. *nSyb-GAL4 > UAS-*Hk^{RNAi} (sni^1/Y)	t_{123} = 6.218	<0.0001
	Holm-Šídák test	sni^1/Y vs. *R23E10-GAL4 > UAS-*Hk^{RNAi} (sni^1/Y)	t_{123} = 6.753	<0.0001
**3c**	Kruskal-Wallis ANOVA			
	Genotype		H_{12} = 301.9	<0.0001
	Dunn's test	*+/Y* vs. sni^1/Y	z = 7.950	<0.0001
	Dunn's test	*+/Y* vs. *nSyb-GAL4* (sni^1/Y)	z = 5.716	<0.0001
	Dunn's test	*+/Y* vs. *R23E10-GAL4* (sni^1/Y)	z = 6.538	<0.0001
	Dunn's test	*+/Y* vs. *UAS-sni* (sni^1/Y)	z = 0.4817	>0.9999
	Dunn's test	*+/Y* vs. *nSyb-GAL4 > UAS-sni* (sni^1/Y)	z = 2.562	0.1250
	Dunn's test	*+/Y* vs. *R23E10-GAL4 > UAS-sni* (sni^1/Y)	z = 2.568	0.1227
	Dunn's test	*+/Y* vs. *UAS-AOX* (sni^1/Y)	z = 4.096	0.0005
	Dunn's test	*+/Y* vs. *nSyb-GAL4 > UAS-AOX* (sni^1/Y)	z = 0.8611	>0.9999
	Dunn's test	*+/Y* vs. *R23E10-GAL4 > UAS-AOX* (sni^1/Y)	z = 1.922	0.6558
	Dunn's test	*+/Y* vs. *UAS-*Hk^{RNAi} (sni^1/Y)	z = 4.916	<0.0001
	Dunn's test	*+/Y* vs. *nSyb-GAL4 > UAS-*Hk^{RNAi} (sni^1/Y)	z = 2.831	0.0556
	Dunn's test	*+/Y* vs. *R23E10-GAL4 > UAS-*Hk^{RNAi} (sni^1/Y)	z = 1.054	>0.9999
**4b, τ_{fast}**	Unpaired *t*-test	*+/Y* vs. sni^1/Y	t_{31} = 2.951	0.0060
**4b, τ_{slow}**	Mann-Whitney test	*+/Y* vs. sni^1/Y	U = 74	0.0253
**4d, τ_{fast}**	One-way repeated-measures ANOVA			
	Time		$F_{1.306,16.98}$ = 6.757	0.0133
	Holm-Šídák test	0 min vs. 10 min	t_{13} = 3.300	0.0171
	Holm-Šídák test	0 min vs. 20 min	t_{13} = 2.537	0.0248
	Holm-Šídák test	0 min vs. 30 min	t_{13} = 3.114	0.0171
**4d, τ_{slow}**	One-way repeated-measures ANOVA			
	Time		$F_{2.243,29.15}$ = 6.304	0.0041
	Holm-Šídák test	0 min vs. 10 min	t_{13} = 3.028	0.0228
	Holm-Šídák test	0 min vs. 20 min	t_{13} = 2.790	0.0228
	Holm-Šídák test	0 min vs. 30 min	t_{13} = 3.150	0.0228
**4f, τ_{fast}**	Mixed-effects model			
	Time		$F_{1.493,13.44}$ = 12.67	0.0015
	Holm-Šídák test	0 min vs. 10 min	t_{10} = 3.961	0.0054
	Holm-Šídák test	0 min vs. 20 min	t_{10} = 4.382	0.0041
	Holm-Šídák test	0 min vs. 30 min	t_{7} = 3.304	0.0131
**4f, τ_{slow}**	Mixed-effects model			
	Time		$F_{2.361,20.46}$ = 0.076	0.0010
	Holm-Šídák test	0 min vs. 10 min	t_{10} = 2.537	0.0295
	Holm-Šídák test	0 min vs. 20 min	t_{8} = 4.144	0.0075
	Holm-Šídák test	0 min vs. 30 min	t_{6} = 4.108	0.0090
**4g, τ_{fast}**	Kruskal-Wallis ANOVA			
	Genotype and/or treatment		H_{6} = 42.21	<0.0001
	Dunn's test	*+/+* vs. sleep deprivation (*+/+*)	z = 2.865	0.0250
	Dunn's test	*+/+* vs. 0.15% (v/v) methyl acetate (*+/+*)	z = 1.121	>0.9999
	Dunn's test	*+/+* vs. 4-ONE (*+/+*)	z = 4.479	<0.0001
	Dunn's test	*+/+* vs. 4-HNE (*+/+*)	z = 0.323	>0.9999
	Dunn's test	*+/+* vs. 4-ONE (Hk^1/Hk^1;*R23E10-GAL4 > UAS-Hk*)	z = 4.763	<0.0001
	Dunn's test	*+/+* vs. 4-ONE (Hk^1/Hk^1;*R23E10-GAL4 > UAS-*Hk^{K289M})	z = 0.719	>0.9999
**4g, τ_{slow}**	Kruskal-Wallis ANOVA			
	Genotype and/or treatment		H_{6} = 34.29	<0.0001
	Dunn's test	*+/+* vs. sleep deprivation (*+/+*)	z = 3.037	0.0143
	Dunn's test	*+/+* vs. 0.15% (v/v) methyl acetate (*+/+*)	z = 0.930	>0.9999
	Dunn's test	*+/+* vs. 4-ONE (*+/+*)	z = 4.478	<0.0001
	Dunn's test	*+/+* vs. 4-HNE (*+/+*)	z = 0.896	>0.9999
	Dunn's test	*+/+* vs. 4-ONE (Hk^1/Hk^1;*R23E10-GAL4 > UAS-Hk*)	z = 3.802	0.0009

	Dunn's test	+/+ vs. 4-ONE (*Hk[1]/Hk[1];R23E10-GAL4 > UAS-Hk*^{K289M})	z = 0.501	>0.9999
**5a**	Two-way repeated-measures ANOVA			
	Current		$F_{1.835,34.86}$ = 57.91	<0.0001
	Genotype		$F_{1,19}$ = 19.86	0.0003
	Current × genotype		$F_{15,2855}$ = 4.933	<0.0001
**5b**	Two-way repeated-measures ANOVA			
	Current		$F_{1.856,20.42}$ = 29.80	<0.0001
	Illumination		$F_{1,11}$ = 6.907	0.0235
	Current × illumination		$F_{15,65}$ = 2.755	0.0008
**5c**	Two-way repeated-measures ANOVA			
	Current		$F_{1.861,37.23}$ = 51.62	<0.0001
	4-ONE		$F_{1,20}$ = 0.015	0.9052
	Current × 4-ONE		$F_{15,300}$ = 0.670	0.7846
**6b, τ_{fast}**	Mixed-effects model			
	Time		$F_{2.523,48.77}$ = 14.68	<0.0001
	Holm-Šídák test	0 min vs. 10 min	t_{22} = 4.100	0.0009
	Holm-Šídák test	0 min vs. 30 min	t_{20} = 1.069	0.2979
	Holm-Šídák test	0 min vs. 40 min	t_{16} = 4.770	0.0006
**6b, τ_{slow}**	Mixed-effects model			
	Time		$F_{2.635,50.95}$ = 7.047	0.0008
	Holm-Šídák test	0 min vs. 10 min	t_{22} = 2.890	0.0169
	Holm-Šídák test	0 min vs. 30 min	t_{20} = 0.333	0.7425
	Holm-Šídák test	0 min vs. 40 min	t_{16} = 3.479	0.0093
**6d, τ_{fast}**	Mixed-effects model			
	Time		$F_{1.102,19.10}$ = 9.404	0.0053
	Holm-Šídák test	0 min vs. 10 min	t_{21} = 4.450	0.0007
	Holm-Šídák test	0 min vs. 30 min	t_{20} = 0.191	0.8506
	Holm-Šídák test	0 min vs. 40 min	t_{11} = 2.598	0.0489
**6d, τ_{slow}**	Mixed-effects model			
	Time		$F_{1.473,22.09}$ = 10.91	0.0012
	Holm-Šídák test	0 min vs. 10 min	t_{19} = 3.759	0.0040
	Holm-Šídák test	0 min vs. 30 min	t_{18} = 0.645	0.5270
	Holm-Šídák test	0 min vs. 40 min	t_8 = 2.756	0.0490

Supplementary Table 2. Statistical analyses of data in Extended Data Figures 1–8.

Figure (ED)	Statistical test	Pairwise comparison	Test statistic	*P*
**1a**	Two-way repeated-measures ANOVA			
	Zeitgeber time		$F_{47,7003}$ = 169.5	<0.0001
	Genotype		$F_{3,149}$ = 119.9	<0.0001
	Zeitgeber time × Genotype		$F_{141,7003}$ = 14.44	<0.0001
	Holm-Šídák test	+/Y vs. *sni*^{1}/Y	t_{149} = 10.48	<0.0001
	Holm-Šídák test	+/Y vs. *nSyb-GAL4 > UAS-sni (sni*^{1}*/Y)*	t_{149} = 5.443	<0.0001
	Holm-Šídák test	+/Y vs. *R23E10-GAL4 > UAS-sni (sni*^{1}*/Y)*	t_{149} = 5.292	<0.0001
	Holm-Šídák test	*sni*^{1}/Y vs. *nSyb-GAL4 > UAS-sni (sni*^{1}*/Y)*	t_{149} = 16.84	<0.0001
	Holm-Šídák test	*sni*^{1}/Y vs. *R23E10-GAL4 > UAS-sni (sni*^{1}*/Y)*	t_{149} = 16.63	<0.0001
**1b**	Kruskal-Wallis ANOVA			
	Genotype		H_{12} = 238.1	<0.0001
	Dunn's test	+/Y vs. *sni*^{1}/Y	z = 8.363	<0.0001
	Dunn's test	+/Y vs. *nSyb-GAL4 (sni*^{1}*/Y)*	z = 6.268	<0.0001
	Dunn's test	+/Y vs. *R23E10-GAL4 (sni*^{1}*/Y)*	z = 6.915	<0.0001
	Dunn's test	+/Y vs. *UAS-sni (sni*^{1}*/Y)*	z = 0.8119	>0.9999
	Dunn's test	+/Y vs. *nSyb-GAL4 > UAS-sni (sni*^{1}*/Y)*	z = 1.118	>0.9999
	Dunn's test	+/Y vs. *R23E10-GAL4 > UAS-sni (sni*^{1}*/Y)*	z = 0.4924	>0.9999
	Dunn's test	+/Y vs. *UAS-AOX (sni*^{1}*/Y)*	z = 5.065	<0.0001
	Dunn's test	+/Y vs. *nSyb-GAL4 > UAS-AOX (sni*^{1}*/Y)*	z = 1.162	>0.9999
	Dunn's test	+/Y vs. *R23E10-GAL4 > UAS-AOX (sni*^{1}*/Y)*	z = 2.507	0.1462
	Dunn's test	+/Y vs. *UAS-Hk*^{RNAi} *(sni*^{1}*/Y)*	z = 3.956	0.0009
	Dunn's test	+/Y vs. *nSyb-GAL4 > UAS-Hk*^{RNAi} *(sni*^{1}*/Y)*	z = 3.049	0.0276
	Dunn's test	+/Y vs. *R23E10-GAL4 > UAS-Hk*^{RNAi} *(sni*^{1}*/Y)*	z = 1.208	>0.9999
**1c**	Kruskal-Wallis ANOVA			
	Genotype		H_{12} = 190.6	<0.0001
	Dunn's test	+/Y vs. *sni*^{1}/Y	z = 8.627	<0.0001
	Dunn's test	+/Y vs. *nSyb-GAL4 (sni*^{1}*/Y)*	z = 6.376	<0.0001
	Dunn's test	+/Y vs. *R23E10-GAL4 (sni*^{1}*/Y)*	z = 7.057	<0.0001
	Dunn's test	+/Y vs. *UAS-sni (sni*^{1}*/Y)*	z = 1.876	0.7271
	Dunn's test	+/Y vs. *nSyb-GAL4 > UAS-sni (sni*^{1}*/Y)*	z = 0.2178	>0.9999
	Dunn's test	+/Y vs. *R23E10-GAL4 > UAS-sni (sni*^{1}*/Y)*	z = 1.136	>0.9999
	Dunn's test	+/Y vs. *UAS-AOX (sni*^{1}*/Y)*	z = 5.202	<0.0001
	Dunn's test	+/Y vs. *nSyb-GAL4 > UAS-AOX (sni*^{1}*/Y)*	z = 1.382	>0.9999
	Dunn's test	+/Y vs. *R23E10-GAL4 > UAS-AOX (sni*^{1}*/Y)*	z = 2.500	0.1492
	Dunn's test	+/Y vs. *UAS-Hk*^{RNAi} *(sni*^{1}*/Y)*	z = 3.366	0.0091
	Dunn's test	+/Y vs. *nSyb-GAL4 > UAS-Hk*^{RNAi} *(sni*^{1}*/Y)*	z = 2.896	0.0454
	Dunn's test	+/Y vs. *R23E10-GAL4 > UAS-Hk*^{RNAi} *(sni*^{1}*/Y)*	z = 1.442	>0.9999
**1d**	Kruskal-Wallis ANOVA			
	Genotype		H_{12} = 173.9	<0.0001
	Dunn's test	+/Y vs. *sni*^{1}/Y	z = 6.796	<0.0001
	Dunn's test	+/Y vs. *nSyb-GAL4 (sni*^{1}*/Y)*	z = 2.560	0.1256
	Dunn's test	+/Y vs. *R23E10-GAL4 (sni*^{1}*/Y)*	z = 5.298	<0.0001
	Dunn's test	+/Y vs. *UAS-sni (sni*^{1}*/Y)*	z = 2.871	0.0491
	Dunn's test	+/Y vs. *nSyb-GAL4 > UAS-sni (sni*^{1}*/Y)*	z = 4.769	<0.0001
	Dunn's test	+/Y vs. *R23E10-GAL4 > UAS-sni (sni*^{1}*/Y)*	z = 6.535	<0.0001
	Dunn's test	+/Y vs. *UAS-AOX (sni*^{1}*/Y)*	z = 3.299	0.0116
	Dunn's test	+/Y vs. *nSyb-GAL4 > UAS-AOX (sni*^{1}*/Y)*	z = 4.239	0.0003
	Dunn's test	+/Y vs. *R23E10-GAL4 > UAS-AOX (sni*^{1}*/Y)*	z = 3.534	0.0049
	Dunn's test	+/Y vs. *UAS-Hk*^{RNAi} *(sni*^{1}*/Y)*	z = 9.281	<0.0001
	Dunn's test	+/Y vs. *nSyb-GAL4 > UAS-Hk*^{RNAi} *(sni*^{1}*/Y)*	z = 8.585	<0.0001
	Dunn's test	+/Y vs. *R23E10-GAL4 > UAS-Hk*^{RNAi} *(sni*^{1}*/Y)*	z = 7.988	<0.0001
**3a**, τ_{fast}	Mixed-effects model			
	Time		$F_{1.107,11.07}$ = 1.501	0.2499
**3a**, τ_{slow}	Mixed-effects model			
	Time		$F_{1.736,17.36}$ = 0.4867	0.5968
**3a**, I_A	Mixed-effects model			
	Time		$F_{1.640,16.40}$ = 14.56	0.0004
	Holm-Šídák test	0 min vs. 10 min	t_{12} = 5.084	0.0008
	Holm-Šídák test	0 min vs. 20 min	t_{11} = 4.225	0.0028
	Holm-Šídák test	0 min vs. 30 min	t_7 = 4.333	0.0034
**3a**, $I_{non\text{-}A}$	Mixed-effects model			
	Time		$F_{1.869,18.69}$ = 1.090	0.3527
**3a**, R_m	Mixed-effects model			
	Time		$F_{1.847,18.47}$ = 0.409	0.6543
**3a**, τ_m	Mixed-effects model			
	Time		$F_{1.924,17.96}$ = 0.668	0.5196
**3b**, τ_{fast}	Mixed-effects model			

	Time		$F_{1.662,124.09} = 7.164$	0.0054
	Holm-Šídák test	0 min vs. 10 min	$t_{16} = 3.774$	0.0033
	Holm-Šídák test	0 min vs. 30 min	$t_{13} = 0.9755$	0.3471
**3b, τ_{slow}**	Mixed-effects model			
	Time		$F_{1.972,28.59} = 8.419$	0.0014
	Holm-Šídák test	0 min vs. 10 min	$t_{16} = 3.478$	0.0062
	Holm-Šídák test	0 min vs. 30 min	$t_{13} = 0.6259$	0.5422
**3b, I_A**	Mixed-effects model			
	Time		$F_{1.392,20.18} = 12.61$	0.0008
	Holm-Šídák test	0 min vs. 10 min	$t_{16} = 3.710$	0.0024
	Holm-Šídák test	0 min vs. 30 min	$t_{13} = 4.124$	0.0024
**3b, $I_{non\text{-}A}$**	Mixed-effects model			
	Time		$F_{1.266,18.35} = 1.154$	0.3120
**3b, R_m**	Mixed-effects model			
	Time		$F_{1.444,18.05} = 0.6253$	0.4961
**3b, τ_m**	Mixed-effects model			
	Time		$F_{1.503,18.03} = 1.604$	0.2282
**3c**	Mixed-effects model			
	Time		$F_{1.177,17.06} = 4.677$	0.0399
	Holm-Šídák test	0 min vs. 10 min	$t_{16} = 1.725$	0.1039
	Holm-Šídák test	0 min vs. 30 min	$t_{13} = 2.431$	0.0597
**3d**	*F* test			
	I_A activation	H_0: same $V_{0.5}$ and slope factor	$F_{4,482} = 0.7811$	0.5378
	I_A inactivation	H_0: same $V_{0.5}$ and slope factor	$F_{4,710} = 6.209$	<0.0001
**4a, I_A**	Mann-Whitney test	*+/Y* vs. *sni^1/Y*	U = 112	0.4023
**4a, $I_{non\text{-}A}$**	Unpaired *t*-test	*+/Y* vs. *sni^1/Y*	$t_{31} = 0.4900$	0.6276
**4a, R_m**	Unpaired *t*-test	*+/Y* vs. *sni^1/Y*	$t_{25} = 1.055$	0.3014
**4a, τ_m**	Mann-Whitney test	*+/Y* vs. *sni^1/Y*	U = 71	0.5267
**4b, I_A**	Friedman test			
	Time		$\chi^2 = 18.60$	0.0003
	Dunn's test	0 min vs. 10 min	z = 3.513	0.0013
	Dunn's test	0 min vs. 20 min	z = 3.367	0.0023
	Dunn's test	0 min vs. 30 min	z = 3.660	0.0008
**4b, $I_{non\text{-}A}$**	Friedman test			
	Time		$\chi^2 = 6.000$	0.1116
**4b, R_m**	One-way repeated-measures ANOVA			
	Time		$F_{1.315,17.10} = 0.744$	0.4361
**4b, τ_m**	Mixed-effects model			
	Time		$F_{1.203,13.63} = 1.100$	0.3265
**4c, I_A**	Mixed-effects model			
	Time		$F_{1.014,9.130} = 17.11$	0.0024
	Holm-Šídák test	0 min vs. 10 min	$t_{10} = 4.320$	0.0030
	Holm-Šídák test	0 min vs. 20 min	$t_{10} = 5.207$	0.0012
	Holm-Šídák test	0 min vs. 30 min	$t_7 = 3.561$	0.0092
**4c, $I_{non\text{-}A}$**	Mixed-effects model			
	Time		$F_{0.2175,1.958} = 1.258$	0.2067
**4c, R_m**	Mixed-effects model			
	Time		$F_{0.6739,5.840} = 1.125$	0.2942
**4c, τ_m**	Mixed-effects model			
	Time		$F_{1.408,12.20} = 3.424$	0.0783
**5b, τ_{fast}**	One-way repeated-measures ANOVA			
	Time		$F_{1.294,9.060} = 0.257$	0.6841
**5b, τ_{slow}**	Friedman test			
	Time		$\chi^2 = 0.7500$	0.7943

**5b, I_A**	One-way repeated-measures ANOVA			
	Time		$F_{1.579,11.06} = 8.259$	0.0087
	Holm-Šídák test	0 min vs. 10 min	$t_7 = 3.212$	0.0231
	Holm-Šídák test	0 min vs. 30 min	$t_7 = 3.389$	0.0231
**5b, $I_{non\text{-}A}$**	One-way repeated-measures ANOVA			
	Time		$F_{1.091,7.639} = 0.220$	0.6730
**5b, R_m**	One-way repeated-measures ANOVA			
	Time		$F_{1.325,9.277} = 1.501$	0.2615
**5b, τ_m**	One-way repeated-measures ANOVA			
	Time		$F_{1.590,11.13} = 0.1516$	0.8143
**5d, τ_{fast}**	Friedman test			
	Time		$\chi^2 = 11.45$	0.0020
	Dunn's test	0 min vs. 10 min	$z = 2.558$	0.0210
	Dunn's test	0 min vs. 30 min	$z = 0.6396$	>0.9999
**5d, τ_{slow}**	Friedman test			
	Time		$\chi^2 = 16.55$	<0.0001
	Holm-Šídák test	0 min vs. 10 min	$z = 3.624$	0.0006
	Holm-Šídák test	0 min vs. 30 min	$z = 0.2132$	>0.9999
**5d, I_A**	One-way repeated-measures ANOVA			
	Time		$F_{1.425,14.25} = 27.82$	<0.0001
	Holm-Šídák test	0 min vs. 10 min	$t_{10} = 6.482$	0.0001
	Holm-Šídák test	0 min vs. 30 min	$t_{10} = 5.886$	0.0001
**5d, $I_{non\text{-}A}$**	One-way repeated-measures ANOVA			
	Time		$F_{1.626,16.26} = 0.824$	0.4334
**5d, R_m**	Mixed-effects model			
	Time		$F_{1.711,16.25} = 0.480$	0.5984
**5d, τ_m**	Friedman test			
	Time		$\chi^2 = 0.1818$	0.9761
**6a, I_A**	Mixed-effects model			
	Time		$F_{1.948,37.66} = 19.47$	<0.0001
	Holm-Šídák test	0 min vs. 10 min	$t_{22} = 3.414$	0.0025
	Holm-Šídák test	0 min vs. 30 min	$t_{20} = 4.044$	0.0013
	Holm-Šídák test	0 min vs. 40 min	$t_{16} = 5.773$	<0.0001
**6a, $I_{non\text{-}A}$**	Mixed-effects model			
	Time		$F_{0.8893,17.19} = 2.078$	0.1673
**6a, R_m**	Mixed-effects model			
	Time		$F_{2.518,22.66} = 2.838$	0.0688
**6a, τ_m**	Mixed-effects model			
	Time		$F_{1.839,17.47} = 1.258$	0.3058
**6b, I_A**	Mixed-effects model			
	Time		$F_{2.086,36.16} = 13.05$	<0.0001
	Holm-Šídák test	0 min vs. 10 min	$t_{21} = 4.470$	0.0006
	Holm-Šídák test	0 min vs. 30 min	$t_{20} = 4.329$	0.0007
	Holm-Šídák test	0 min vs. 40 min	$t_{11} = 4.524$	0.0009
**6b, $I_{non\text{-}A}$**	Mixed-effects model			
	Time		$F_{1.634,28.33} = 0.545$	0.6240
**6b, R_m**	Mixed-effects model			
	Time		$F_{2.144,31.45} = 8.637$	0.0008
	Holm-Šídák test	0 min vs. 10 min	$t_{18} = 1.701$	0.1062
	Holm-Šídák test	0 min vs. 30 min	$t_{16} = 4.159$	0.0022
	Holm-Šídák test	0 min vs. 40 min	$t_{10} = 4.357$	0.0029
**6b, τ_m**	Mixed-effects model			
	Time		$F_{0.2544,2714} = 3.313$	0.1258
**7c, τ_{fast}**	Friedman test			
	Time		$\chi^2 = 12.25$	0.0009
	Dunn's test	0 min vs. 10 min	$z = 3.250$	0.0023
	Dunn's test	0 min vs. 30 min	$z = 0.5000$	>0.9999
**7c, I_A**	One-way repeated-measures ANOVA			

	Time<br>Holm-Šídák test<br>Holm-Šídák test	 0 min vs. 10 min<br>0 min vs. 30 min	$F_{1.517,10.62} = 8.996$<br>$t_7 = 2.995$<br>$t_7 = 3.378$	0.0075<br>0.0235<br>0.0235
**7c, $I_{non\text{-}A}$**	One-way repeated-measures ANOVA<br>Time		 $F_{1.920,13.44} = 2.719$	 0.1035
**7c, R_m**	Mixed-effects model<br>Time		 $F_{1.762,11.45} = 0.8127$	 0.4532
**7c, τ_m**	Friedman test<br>Time		 $\chi^2 = 2.000$	 0.4861
**7e, τ_{fast}**	Mixed-effects model<br>Time		 $F_{1.534,11.50} = 1.124$	 0.3416
**7e, I_A**	Mixed-effects model<br>Time<br>Dunn's test<br>Dunn's test	 <br>0 min vs. 10 min<br>0 min vs. 30 min	 $F_{1.116,8.370} = 5.110$<br>$t_8 = 2.055$<br>$t_7 = 2.129$	 0.0496<br>0.1365<br>0.1365
**7e, $I_{non\text{-}A}$**	Mixed-effects model<br>Time		 $F_{0.9525,7.144} = 0.8879$	 0.3712
**7e, R_m**	Mixed-effects model<br>Time		 $F_{0.9922,7.441} = 2.891$	 0.1304
**7e, τ_m**	Mixed-effects model<br>Time		 $F_{1.219,9.142} = 1.840$	 0.2109
**7h, τ_{fast}**	Mann-Whitney test	Untreated vs. methylglyoxal (pooled from panels h, j)	U = 6	$<$0.0001
**7h, τ_{slow}**	Mann-Whitney test	Untreated vs. methylglyoxal (pooled from panels h, j)	U = 30	0.0003
**7h, I_A**	Unpaired *t*-test	Untreated vs. methylglyoxal (pooled from panels h, j)	$t_{28} = 1.763$	0.0888
**7h, τ_{fast}**	Wilcoxon test	0 min vs. 20 min	W = 7	0.4375
**7h, τ_{slow}**	Wilcoxon test	0 min vs. 20 min	W = 11	0.1875
**7h, I_A**	Paired *t*-test	0 min vs. 20 min	$t_4 = 1.763$	0.0429
**7j, τ_{fast}**	Paired *t*-test	0 min vs. 20 min	$t_9 = 2.586$	0.0294
**7j, τ_{slow}**	Wilcoxon test	0 min vs. 20 min	W = –47	0.0137
**7j, I_A**	Paired *t*-test	0 min vs. 20 min	$t_9 = 2.765$	0.0219
**8b**	Kruskal-Wallis ANOVA<br>Genotype and/or sleep history<br>Dunn's test<br>Dunn's test	 <br>*+/+* vs. sni^1/Y<br>Rested vs. sleep-deprived	 $H_2 = 7.887$<br>z = 2.197<br>z = 2.613	 0.0194<br>0.0179<br>0.0560
