# Supplementary material for: Sleep pressure accumulates in a voltage-gated lipid peroxidation memory
Source: Nature. 2025 Mar 19;641(8061):232–9. doi: 10.1038/s41586-025-08734-4 (PMC12043502; doi:10.1038/s41586-025-08734-4)
Supplement: Supplementary file 2 — Reporting Summary [file 41586_2025_8734_MOESM2_ESM.pdf]

nature portfolio

Corresponding author(s): Gero Miesenböck

Last updated by author(s): Jan 24, 2025

Reporting Summary

Nature Portfolio wishes to improve the reproducibility of the work that we publish. This form provides structure for consistency and transparency in reporting. For further information on Nature Portfolio policies, see our Editorial Policies and the Editorial Policy Checklist.

Statistics

For all statistical analyses, confirm that the following items are present in the figure legend, table legend, main text, or Methods section.

n/a	Confirmed	
☐	☒	The exact sample size (*n*) for each experimental group/condition, given as a discrete number and unit of measurement
☐	☒	A statement on whether measurements were taken from distinct samples or whether the same sample was measured repeatedly
☐	☒	The statistical test(s) used AND whether they are one- or two-sided<br>*Only common tests should be described solely by name; describe more complex techniques in the Methods section.*
☒	☐	A description of all covariates tested
☐	☒	A description of any assumptions or corrections, such as tests of normality and adjustment for multiple comparisons
☐	☒	A full description of the statistical parameters including central tendency (e.g. means) or other basic estimates (e.g. regression coefficient) AND variation (e.g. standard deviation) or associated estimates of uncertainty (e.g. confidence intervals)
☐	☒	For null hypothesis testing, the test statistic (e.g. *F*, *t*, *r*) with confidence intervals, effect sizes, degrees of freedom and *P* value noted<br>*Give P values as exact values whenever suitable.*
☒	☐	For Bayesian analysis, information on the choice of priors and Markov chain Monte Carlo settings
☒	☐	For hierarchical and complex designs, identification of the appropriate level for tests and full reporting of outcomes
☒	☐	Estimates of effect sizes (e.g. Cohen's *d*, Pearson's *r*), indicating how they were calculated

Our web collection on statistics for biologists contains articles on many of the points above.

Software and code

Policy information about availability of computer code

Data collection	SMALDI-MSI: SMALDIControl 1.3 (TransMIT GmbH) and Tune 2.8 (ThermoFisher)<br>LC-MS2: Tune 2.9 (ThermoFisher) and Thermo XCalibur 4.0.27.19 (ThermoFisher)<br>Sleep behaviour: Trikinetics DAM system<br>Electrophysiology: pCLAMP 10 or 11 (Molecular Devices)<br>Confocal images: Leica Application Suite; summed-intensity projections were computed in Fiji 2.14.0/1.54f
Data analysis	SMALDI-MSI data were analysed in Mirion 3.3.64.23 (TransMIT GmbH) and annotated in bulk structure searches against the COMP_DB database in LIPID MAPS (https://www.lipidmaps.org). Signal intensities and degrees of enrichment of molecular features were analysed in MATLAB R2023b and the Differential Expression Analysis module of LipidSig (https://lipidsig.bioinfomics.org/DE/).<br>LC-MS2 data were annotated using LipidMatch 3.5 (Innovative Omics).<br>Sleep behaviour data were analysed with the Sleep and Circadian Analysis MATLAB Program (SCAMP v3).<br>Electrophysiological data were analysed using version 3.0c of the NeuroMatic package in Igor Pro 8.04 (WaveMetrics).<br>All normality and hypothesis tests were performed in Prism 10.4.1 (GraphPad).

For manuscripts utilizing custom algorithms or software that are central to the research but not yet described in published literature, software must be made available to editors and reviewers. We strongly encourage code deposition in a community repository (e.g. GitHub). See the Nature Portfolio guidelines for submitting code & software for further information.

Data

Policy information about availability of data

All manuscripts must include a data availability statement. This statement should provide the following information, where applicable:

- Accession codes, unique identifiers, or web links for publicly available datasets
- A description of any restrictions on data availability
- For clinical datasets or third party data, please ensure that the statement adheres to our policy

The SMALDI-MSI and LC-MS2 datasets are accessible in METASPACE (https://metaspace2020.eu/project/drosophila and https://metaspace2020.eu/project/drosophila4ONE) and the MassIVE repository (ftp://massive.ucsd.edu/v05/MSV000091767/), respectively. All other data generated and analysed during this study are included in the Source Data file.

Research involving human participants, their data, or biological material

Policy information about studies with human participants or human data. See also policy information about sex, gender (identity/presentation), and sexual orientation and race, ethnicity and racism.

Reporting on sex and gender	n/a
Reporting on race, ethnicity, or other socially relevant groupings	n/a
Population characteristics	n/a
Recruitment	n/a
Ethics oversight	n/a

Note that full information on the approval of the study protocol must also be provided in the manuscript.

Field-specific reporting

Please select the one below that is the best fit for your research. If you are not sure, read the appropriate sections before making your selection.

☒ Life sciences ☐ Behavioural & social sciences ☐ Ecological, evolutionary & environmental sciences

For a reference copy of the document with all sections, see nature.com/documents/nr-reporting-summary-flat.pdf

Life sciences study design

All studies must disclose on these points even when the disclosure is negative.

Sample size	Sample sizes are provided in each figure and extended data figure or its legend.<br>Sample sizes in behavioural experiments were chosen to detect 2-h differences in daily sleep with a power of 0.8.<br>Sample sizes in electrophysiological experiments are based on precedent (Kempf et al., Nature 2019).<br>Sample sizes in lipidomic experiments match those of example datasets in the Differential Expression Analysis module of LipidSig (https://lipidsig.bioinfomics.org/DE/).
Data exclusions	Immobile flies (< 2 beam breaks per 24 h) were excluded from sleep measurements.<br>Voltage-clamp recordings were terminated if the series resistance increased by >20% from baseline or exceeded 50 MΩ.<br>If fits of the slow inactivation time constants of A-type currents were poorly constrained, only the fast inactivation time constants were analysed.
Replication	Results were replicated on different brain sections, flies, or cells across each dataset. All replicates are included in figures and extended data figures.
Randomization	Flies of the correct genotype and sex, as indicated in Methods, were selected randomly for analysis and assigned randomly to treatment groups if treatments were applied (e.g., sleep deprivation).
Blinding	The investigators were not blind to group allocation. Measurements and analyses were automated and/or required the performance of genotype-specific experimental protocols.

Reporting for specific materials, systems and methods

We require information from authors about some types of materials, experimental systems and methods used in many studies. Here, indicate whether each material, system or method listed is relevant to your study. If you are not sure if a list item applies to your research, read the appropriate section before selecting a response.

Materials & experimental systems

n/a	Involved in the study
☐	☒ Antibodies
☐	☒ Eukaryotic cell lines
☒	☐ Palaeontology and archaeology
☐	☒ Animals and other organisms
☒	☐ Clinical data
☒	☐ Dual use research of concern
☒	☐ Plants

Methods

n/a	Involved in the study
☒	☐ ChIP-seq
☒	☐ Flow cytometry
☒	☐ MRI-based neuroimaging

Antibodies

Antibodies used	Mouse monoclonal anti-FLAG M2 antibody (Sigma F1804)<br>Goat anti-Mouse Alexa Fluor 633 antibody (ThermoFisher A-21052)
Validation	The mouse monoclonal anti-FLAG M2 antibody recognizes the artificial epitope DYKDDDDK, has been used in immunofluorescence applications in Drosophila (e.g., Tannan et al., PLoS Genet 2018), and shows no cross-reactivity with endogenous Drosophila proteins in our hands (Fig. 5f).

Eukaryotic cell lines

Policy information about cell lines and Sex and Gender in Research

Cell line source(s)	HEK-293 cells (CRL-1573, American Type Culture Collection)
Authentication	Our sample of HEK-293 cells was not externally authenticated.
Mycoplasma contamination	Our sample of HEK-293 cells was not tested routinely for mycoplasma infection.
Commonly misidentified lines (See ICLAC register)	No commonly misidentified cell lines were used in this study.

Animals and other research organisms

Policy information about studies involving animals; ARRIVE guidelines recommended for reporting animal research, and Sex and Gender in Research

Laboratory animals	In all experiments except those involving the sni1 mutation and all relevant controls (see below), females aged 2–6 days after eclosion were used. These experimental flies were heterozygous for all transgenes and homozygous for either a wild-type or mutant (Hk1) Hyperkinetic allele. Transgenes included R23E10-GAL4 or Dh31-GAL4 and UAS–mCD8::GFP, UAS–myr-MS6T2, UAS-Hk, and/or UAS–HkK289M.<br>The function of the X-linked sniffer gene was studied in males aged 2–6 days after eclosion. Hemizygous carriers of the sni1 allele coexpressed UAS–mCD8::GFP, UAS–sni, UAS–AOX, or UAS–HkRNAi (47805GD) transgenes under the control of nSyb–GAL4 or R23E10-GAL4.<br>In the HkFLAG strain, the endogenous Hyperkinetic locus encodes an in-frame fusion with an N-terminal FLAG epitope.
Wild animals	No wild animals were used in this study.
Reporting on sex	Male flies were used in behavioural and electrophysiological analyses of sniffer mutants to facilitate the synthesis of the desired genotypes (the sniffer gene is X-linked). Female flies were used in all other experiments because of their larger body size.
Field-collected samples	No field-collected samples were used in this study.
Ethics oversight	Ethical approval was not required for this study.

Note that full information on the approval of the study protocol must also be provided in the manuscript.

Plants

Seed stocks	n/a
Novel plant genotypes	n/a
Authentication	n/a
